# Exploring deep learning and hybrid approaches in molecular subgrouping and prognostic-related genetic signatures of medulloblastoma

**DOI:** 10.1186/s41016-025-00405-7

**Published:** 2025-09-15

**Authors:** Yanong Li, Hailong Liu, Yawei Liu, Jane Li, Hiro Hiromichi Suzuki, Yaou Liu, Jiang Tao, Xiaoguang Qiu

**Affiliations:** 1 Department of Radiation Oncology, Beijing Tiantan Hospital, Capital Medical University, Beijing, 100070 China; 2 Department of Radiology, Beijing Tiantan Hospital, Capital Medical University, Beijing, 100070 China; 3 School of Basic Medical Sciences, Capital Medical University, Beijing, 100069 China; 4 Department of Radiology, Memorial Sloan Kettering Cancer Center, New York, 10065 USA; 5 Division of Brain Tumor Translational Research, National Cancer Center Research Institute, 5-1-1 Tsukiji, Chuo-Ku, Tokyo, 104-0045 Japan; 6 Department of Neurosurgery, Beijing Tiantan Hospital, Capital Medical University, Beijing, 100070 China

**Keywords:** Medulloblastoma subgroups, MRI, Deep learning, Risk stratification, Convolutional neural network

## Abstract

**Background:**

Deep learning (DL) based on MRI of medulloblastoma enables risk stratification, potentially aiding in therapeutic decisions. This study aims to develop DL models that identify four medulloblastoma molecular subgroups and prognostic-related genetic signatures.

**Methods:**

This retrospective study enrolled 325 patients for model development and an independent external validation cohort of 124 patients, totaling 449 MB patients from 2 medical institutes. Consecutive patients with newly diagnosed MB at MRI (T1-weighted, T2-weighted, and contrast-enhanced T1-weighted) at two medical institutes between January 2015 and June 2023 were identified. Two-stage sequential DL models were designed—MB-CNN that first identifies wingless (WNT), sonic hedgehog (SHH), Group 3, and Group 4. Further, prognostic-related genetic signatures using DL models (MB-CNN_TP53/MYC/Chr11) were developed to predict TP53 mutation, MYC amplification, and chromosome 11 loss status. A hybrid model combining MB-CNN and conventional data (clinical information and MRI features) was compared to a logistic regression model constructed only with conventional data. Four-classification tasks were evaluated with confusion matrices (accuracy) and two-classification tasks with ROC curves (area under the curve (AUC)).

**Results:**

The datasets comprised 449 patients (mean age ± SD at diagnosis, 13.55 years ± 2.33, 249 males). MB-CNN accurately classified MB subgroups in the external test dataset, achieving a median accuracy of 77.50% (range in 76.29% to 78.71%). MB-CNN_TP53/MYC/Chr11 models effectively predicted signatures (AUC of TP53 in SHH: 0.91, MYC amplification in Group 3: 0.87, chromosome 11 loss in Group 4: 0.89). The accuracy of the hybrid model outperformed the logistic regression model (82.20% vs. 59.14%, *P* = .009) and showed comparable performance to MB-CNN (82.20% vs. 77.50%, *P* = 0.105).

**Conclusion:**

MRI-based DL models allowed identification of the molecular medulloblastoma subgroups and prognostic-related genetic signatures.

**Supplementary Information:**

The online version contains supplementary material available at 10.1186/s41016-025-00405-7.

## Background

Medulloblastoma (MB), the most prevalent malignant pediatric brain tumor in the posterior fossa [1], is molecularly classified into four subgroups under the 2021 WHO framework: wingless (WNT), sonic hedgehog (SHH), Group 3 (G3), and Group 4 (G4) [1, 2]. Prognosis varies significantly across subgroups, with WNT tumors demonstrating > 90% 5-year survival, SHH subgroups exhibiting TP53 mutation-dependent heterogeneity, and MYC-amplified G3/G4 cases showing aggressive behavior and poor therapeutic response. This molecular stratification underpins current risk-adapted clinical management strategies [3–11].

Deep learning (DL) has revolutionized preoperative molecular prediction in gliomas, enabling accurate identification of biomarkers such as MGMT promoter methylation and IDH mutations. While preliminary DL applications in MB achieved 85% subgroup classification accuracy [12, 13], critical gaps persist in current MB prognostic models, including insufficient cohort sizes for robust generalization, exclusion of high-risk genetic signatures (TP53 mutations, MYC amplification, chromosome 11 loss) in risk stratification frameworks, and underutilized opportunities to enhance predictive accuracy through multimodal clinical-MRI data integration [14, 15].

To address these limitations, we develop a dual DL framework integrating preoperative MRI with methylation/NGS data. First, MB-CNN classifies tumors into molecular subgroups. Second, prognostic-related genetic signatures using DL models predict high-risk genetic signatures (MB-CNN_TP53, MB-CNN_MYC, MB-CNN_Chr11) specific to each subgroup. Finally, a hybrid model combines radiomic features, conventional imaging biomarkers, and clinical variables to optimize prognostic accuracy. This approach bridges the translational gap between molecular subgroup classification and actionable genetic risk assessment.

##  Methods

### Study design and population

**Fig. 1:**
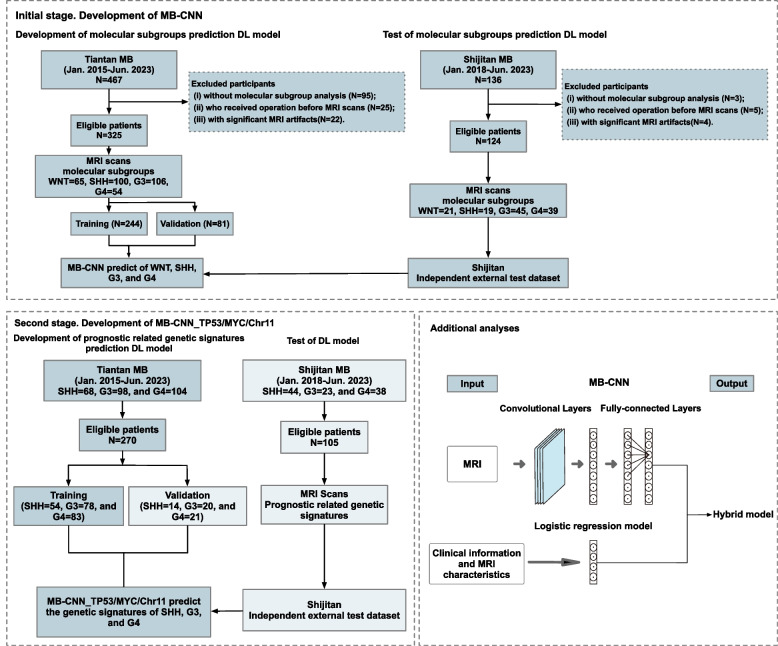
The flowchart of overall study

This study comprised two sequential stages. In the initial stage, a total of 449 patients with MB were enrolled, including 325 patients from Beijing Tiantan Hospital used for model development and 124 patients from Beijing Shijitan Hospital serving as an independent external validation cohort to assess generalizability. In the second stage, a total 375 patients were selected from the first-stage groups with genetic signature information to construct further DL models to predict prognostic related genetic signatures, TP53 mutation (MB-CNN_TP53), MYC amplification (MB-CNN_MYC), and chromosome 11 loss (MB-CNN_Chr11), within the SHH, G3, and G4 subgroups. Furthermore, additional analyses for the hybrid model were performed. Figure 1 shows the overall study flowchart. The total patient’s group with MB in two independent medical institutes is shown in Method S1.

This multicenter retrospective study was approved by the Institutional Review Board (ethics committee approval no. 82172608), with consent waived due to minimal risk. The inclusion criteria were as follows: (i) All patients diagnosed with MB confirmed between January 2015 and June 2023 and (ii) necessary preoperative MR scans including axial T2-weighted (T2W), T1-weighted (T1W), and contrast-enhanced T1-weighted (CE-T1W) sequences. The exclusion criteria were as follows: (i) Patients who lack the molecular subgroup and prognostic-related genetic data, (ii) patients with prior neurosurgical procedures before MR scans, and (iii) MR images with severe artifacts affecting radiological assessment.

### Imaging preprocessing

Brain MR images were performed with at 1.5- or 3.0-T magnet (Philips Healthcare, Siemens Healthineers, GE Healthcare, and Toshiba Medical Systems, USA). The MRI scanning details are in Method S2. Figure S1 shows the proportion of manufacturers across datasets. Preprocessing initiated with conversion to the NIfTI format (FSLv6.0, https://fsl.fmrib.ox.ac.uk/fsl/fslwiki/) for data consistency, followed by spatial resampling and intensity normalization within a range of − 1 to 1 to reduce variability across scanners and improve comparability. Although MRI scans were acquired using different field strengths (1.5 T and 3.0 T) and manufacturers (Philips, Siemens, GE, Toshiba), these preprocessing steps aimed to mitigate inter-scanner differences. N4 bias field correction was not applied in this study but will be considered in future work to further reduce intensity nonuniformity caused by magnetic field inhomogeneities. Method S5 contains comprehensive explanations of these methodological refinements to ensure data consistency and dependability across MR systems.

### Blinded tumor segmentation for ground truth generation

We integrated methylation arrays, next-generation sequencing technologies (whole genome sequencing (WGS) and comparative genomic hybridization (CGH)), to accurately identify four molecular subgroups (presented in Method S4) [16, 17].

For medical image segmentation, the U-Net architecture was selected for its proven effectiveness[18]. Its encoder-decoder design extracts high-dimensional features via convolutional and max pooling layers, while up-convolution layers reconstruct segmentation maps for precise tumor identification.

The segmentation model was developed in the development dataset of 325 patients’ MR scans (T1W, CE-T1W, T2W), split into 80% training (n = 260), 10% validation (*n* = 33), and 10% testing (*n* = 32). Training of the U-Net model was conducted on the PyTorch (https://pytorch.org/) over 100 epochs using the Adam optimizer with a cosine decay learning rate schedule. The Dice loss function serves as the performance metric, with the model’s effectiveness periodically assessed via the Dice score on the validation dataset.

To establish a robust ground truth for the DL model, two neuroradiologists (Y. N. L. and D. C., > 6 years of experience) independently reviewed the segment results of U-Net model. Low-quality images were resegmented using ITK-SNAP (version 3.8.0, http://www.itksnap.org) and refined by a senior neuroradiologist (Y. O. L., 20 years of experience). Overlapping-agreed regions served as the final ground truth for model development.

### Conventional MR characteristics evaluation

Two experienced neuroradiologists (Y. N. L. and D. C.) independently evaluated conventional MR image characteristics across all datasets, including T1W foci hyperintensity, T2W foci hypointensity, and levels of enhancement on CE-T1W images (presented in Method S3). The agreement between readers was excellent (kappa scores: 0.842–0.864, *P* < 0.001). Discrepancies were adjudicated by a senior neuroradiologist (Y. O. L.).

### DL models training and validation

In this study, we developed MB-CNN and MB-CNN_TP53/MYC/Chr11 based on preoperative MR images (T1W, CE-T1W, and T2W). For medical image segmentation, the U-Net architecture was selected for its proven effectiveness[19]. Its encoder-decoder design extracts high-dimensional features via convolutional and max pooling layers, while up-convolution layers reconstruct segmentation maps for precise tumor identification. U-Net was used specifically for the segmentation task due to its skip connections enabling detailed spatial localization, while ResNet-50 was chosen for the classification task because its deep residual learning framework facilitates effective high-level feature extraction and robust classification performance. The ResNet-50 architecture incorporates a 50-layer deep structure designed to overcome the vanishing gradient issue common in extensive neural networks. Its residual blocks enhance gradient propagation throughout the network, enabling the efficient training of deeper neural models.

During the initial stage, for the MB-CNN project, ResNet-50 classified tumor molecular subgroups. Our study leveraged a dataset encompassing MR images from 325 patients, identical to the cohort used for segmentation model training. This dataset was methodically partitioned into training and validation datasets following a 3:1 ratio (244:81), ensuring a randomized distribution. Additionally, a set of 124 independent external patients was designated as the test dataset. Each dataset entry encompasses a tumor segmentation map derived from the original MR scans, facilitated by the corresponding U-Net model. The strategic dataset segmentation ensured equitable representation across the four molecular subgroups.

When the MB-CNN predicts the direct output result of WNT, and when the model predicts SHH, G3, or G4 subgroups, the prediction process will enter the second stage—three specialized two-class DL models (MB-CNN_TP53, MB-CNN_MYC, and MB-CNN_Chr11), based on ResNet-50 to assess prognostic genetic signatures. The models processed raw MR images and tumor segmentation maps produced by U-Net. Prior to final classification through a fully connected layer, the images were resized to dimensions of 256 × 256 × 3 and underwent convolutional processing within the ResNet-50 framework. Subsequently, our model training utilized prognostic-related genetic signatures status from SHH (*n* = 54), G3 (*n* = 78), and G4 (*n* = 83) patient diagnoses. For validation, the model employed prognostic-related genetic signatures identified within the validation dataset (SHH: *n* = 14, G3: *n* = 20, G4: *n* = 21), with independent external test data serving for comprehensive testing (SHH: *n* = 44, G3: *n* = 23, G4: *n* = 38).

The classification model underwent 10 intensive training cycles on the PyTorch (https://pytorch.org/) to enhance its accuracy in classification and prediction tasks. PyTorch was chosen for its support of dynamic computational graphs and GPU acceleration, essential for handling the ResNet-50 architecture and large datasets. To address class imbalance, the focal loss function prioritized hard-to-classify instances, enhancing sensitivity to underrepresented classes. The Adam optimizer adjusted learning rates for efficient convergence, refined by a cosine decay schedule.

To evaluate the model’s generalizability and robustness, an independent dataset of 124 patients was tested, providing insights into the model’s real-world applicability. For four-class classification, the MB-CNN model outputs a four-element vector of probabilities, each representing the likelihood of the input belonging to a specific category, ensured by the softmax activation function (Eq*. *1).

1 $$Softmax\;\left(Z_i\right)=\frac{e^{z_i}}{\sum_j\;e^{z_j}}$$

Here, ${\text{Z}}_{\text{i}}$ is the value of the $\text{i}$ ^th^ element in vector $\text{Z}$, and the denominator is the sum of applying the ${\text{e}}^{\text{x}}$ function to all elements. Here, $\text{e}$ is the base of the natural logarithm, approximately equal to 2.71828.

In binary classification tasks, the model simplifies its output to a single probability value, indicating the likelihood of the input belonging to the “positive” class, a method that streamlines the output for binary tasks while maintaining predictive reliability.

**Fig. 2:**
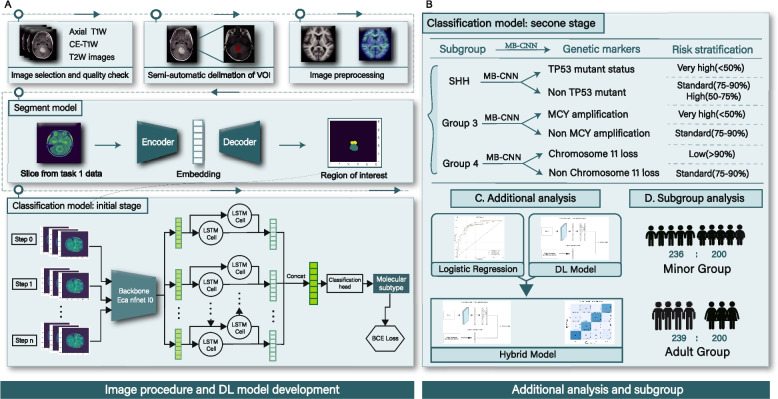
**A** The segmentation and initial stage classification architectures (MB-CNN) of DL model. **B** The performance of the second stage DL model (MB-CNN_TP53/MYC/Chr11) in stratifying patients with MB into different molecular subgroups (SHH, Group 3, and Group 4). **C** The additional analyses of this study (conventional clinical data were used to construct a logistic regression model, and logistic regression model combined with MB-CNN output was used to construct a hybrid model). **D** The complementary sets of subgroup analyses (juvenile group vs. adult group)

The DL model segmentation and classification architectures are shown in Fig. 2A. Further, we independently tested it in the independent external test dataset. Methods S6 and ***S7*** provide the training details of the segmentation and classification models.

### DL model evaluation

The predictive accuracy of the MB-CNN was rigorously assessed using a confusion matrix against the ground truth. The model produced probability scores for each subgroup, facilitating a probabilistic interpretation of results. For classification purposes, each instance was assigned to the molecular subgroup corresponding to the highest probability score. Accuracy and recall for each class were derived from the confusion matrix, offering insights into the model’s performance on a per-class basis. These metrics were complemented by precision and the F1 score, which are particularly informative in scenarios of class imbalance, providing a balanced view of the model’s predictive performance.

The predictive accuracy of the second stage DL model (MB-CNN_TP53/MYC/Chr11) was assessed against ground truth via receiver operating characteristic (ROC) analysis. The model generated continuous probability scores for each subgroup. The optimal cutoff value for classifying subgroups was determined in the training dataset based on the Youden index, which maximizes the combined sensitivity and specificity. Subsequently, the molecular subgroup designation was assigned based on the highest predicted probability. The model’s performance was evaluated using standard metrics, including the area under the ROC curve (AUC), accuracy, sensitivity, and specificity. Further, to ensure a robust evaluation, an independent external dataset comprising 105 patient samples (SHH: *n* = 44, G3: *n* = 23, G4: *n* = 38) was utilized for testing. This external test step is critical for assessing the model’s generalization capability and reliability in real-world scenarios, extending beyond the initial training and validation datasets.

### Additional analyses for clinical applicability

To further elucidate the potential application of the DL model in diverse clinical scenarios, we conducted two complementary sets of additional analyses. First, we constructed a logistic regression model using clinical information (age and gender) and conventional MR characteristics as a baseline for comparison. Subsequently, we built a hybrid model using a logistic regression model incorporating the outputs of the MB-CNN. Ultimately, these analyses aim to evaluate the standalone performance of the MB-CNN. Additionally, they seek to assess the potential enhancive value provided by integrating ancillary inputs, such as clinical data and MR characteristics, into the MB-CNN framework. Finally, we divided the independent test dataset into adult group (> 18 years, *n* = 26) and juvenile group (≤ 18 years, *n* = 98) according to age to compare MB-CNN and MB-CNN_TP53/MYC/Chr11. The models’ performance was evaluated using a confusion matrix (accuracy, recall, precision, and F1 score) and ROC curve (AUC, accuracy, sensitivity, and specificity).

### Statistical analysis

We used the Statistical Package for the Social Sciences (SPSS) software (version 22, IBM, USA) and Python (version 3.6, http://www.python.org) for statistical analyses. Categorical variables were displayed as frequency and percentages and tested using Pearson’s chi-squared test or Fisher’s exact test. Continuous variables were displayed as mean and standard deviation (SD) and were tested using a two-sample *t*-test or Mann–Whitney *U*-test (the choice between the *t*-test and Mann–Whitney *U*-test was based on normality tests). A two-sided *P* < 0.05 was considered statistically significant. Method S8 shows more details of the statistical analysis.

## Results

### Patient characteristics

Of the 449 patients, 325 were used for model training and internal validation, and 124 patients constituted an external test dataset, as detailed in Table 1. Statistical analysis showed no significant differences in age (*P* = 0.13) or gender (*P* = 0.28) distributions across molecular subgroups. Our study cohort consecutively included 449 patients diagnosed with MB, comprising 249 males and 200 females [mean age = 13.63 ± 2.47]. The age distribution revealed three distinct groups: 14 patients younger than 3 years [mean age = 1.86 ± 1.06], 243 patients aged 3 to 18 years [mean age = 11.30 ± 3.12], and 68 adults aged 18 to 49 years [mean age = 28.67 ± 4.33]. Detailed demographic information, genetic profiling, and MR characteristics of the study population are provided in Table 1.

**Table 1:** Demographic and MR features of the training (*n* = 244), validation (*n* = 81), and independent external test dataset (*n* = 124) presented separately

	**Development set**								**Independent external test dataset**			
	Training dataset (*n* = 244)				Validation dataset (*n* = 81)				(*n* = 124)			
**Molecular subgroups**	WNT	SHH	G3	G4	WNT	SHH	G3	G4	WNT	SHH	G3	G4
**Count, *n***	45	76	78	45	20	24	28	9	21	19	45	39
**Demographic**												
Age (mean ± SD), y	14.42 ± 1.33				13.11 ± 2.47				12.53 ± 3.21			
Gender, *n*												
Female-male	22:23	40:36	29:49	12:33	8:12	15:9	8:20	2:7	9:12	10:19	28:17	17:12
**SHH** TP53 (mutant-wild)	43:33				10:14				11:8			
**G3** MYC (amplification-non-amplification)	36:42				12:16				19:26			
**G4** Chromosome 11 (loss-retain)	18:17				3:6				22:17			
**Conventional MRI features**												
Cerebellar origin (yes–no)	3:42	71:5	9:69	3:42	2:18	19:5	5:23	3:6	2:19	13:6	6:39	3:36
Brainstem involvement (yes–no)	13:32	10:66	61:17	39:6	7:13	9:15	10:18	3:6	6:15	4:15	29:16	25:14
T1W images foci-hyperintensity (present-absent)	29:26	36:40	40:38	25:20	11:9	11:13	11:17	5:4	11:10	12:7	24:21	22:17
Enhancement degree on CE-T1W images (mild-obvious)	25:20	44:32	47:31	24:21	11:9	10:14	12:16	3:6	9:12	7:12	31:14	17:22
Enhancement appearance (homogeneous-heterogeneous)	17:28	36:40	33:45	18:27	12:8	6:18	13:15	2:7	7:14	8:11	17:28	13:26
T2W images foci-hypointensity (present-absent)	14:31	49:27	55:33	31:14	11:9	17:7	16:14	6:3	12:9	13:18	22:23	19:20
Hemorrhage (present-absent)	4:41	22:54	18:60	28:17	12:8	19:5	19:9	5:4	7:14	15:13	33:12	21:17
Cyst or necrosis (present-absent)	14:31	31:45	43:35	32:13	9:11	18:6	13:15	3:6	5:16	17:11	30:15	19:20
Dissemination/metastasis (yes–no)	12:33	18:58	23:55	19:28	5:15	7:17	8:20	2:7	4:17	5:23	12:33	5:34
Tumor volume (cm^3^)	284.12 ± 13.22	198.11 ± 34.31	254.69 ± 35.51	336.80 ± 16.79	190.87 ± 34.21	223.01 ± 28.28	201.47 ± 21.55	240.38 ± 31.7	235.00 ± 12.70	191.10 ± 19.48	276.63 ± 23.14	304.26 ± 17.88
*WNT* wingless, *SHH* sonic hedgehog, *G3* Group 3, and *G4* Group 4 *P*-values for demographic and imaging features were calculated to assess intergroup differences. No significant differences were observed in age or gender distributions across molecular subgroups												

### Evaluation of different algorithmic models

In this investigation, we conducted a comparative analysis of four distinct algorithms by training each on a dataset and then evaluating their respective performances on a validation dataset. The algorithms tested were VGG, U-Net, GoogLeNet, and ResNet-50. Based on the precision values with 95% confidence intervals (CIs), the following results were observed: VGG achieved a precision of 65.03% [95% *CI*: 47.28%–71.66%], U-Net reached a precision of 72.00% [95% *CI*: 62.78%–82.33%], GoogLeNet yielded a precision of 69.06% [95% *CI*: 55.43%–79.87%], and ResNet-50 led with a precision of 78.05% [95% *CI*: 69.00%–86.33%]. Given these outcomes, ResNet-50 emerged as the most proficient model in terms of classification accuracy and was subsequently chosen as the final model for our classification tasks. Table S1 and Fig. S2presented additional details.

### Results of initial stage: molecular subgrouping by MB-CNN

The Dice score of MB-CNN was 0.91 ± 0.13. In the validation dataset, MB-CNN demonstrated notable accuracy in differentiating the four molecular subgroups of MB: WNT, SHH, G3, and G4. Comprehensive evaluation indicated that the model achieved an accuracy ranging from 74.68% to 77.84% and a precision rate between 73.85% and 78.90%. The model also showed a reliable range for Recall and F1 score, approximately spanning from 74.69% to 77.81% and 63.60% to 80.00%, respectively.

**Fig. 3:**
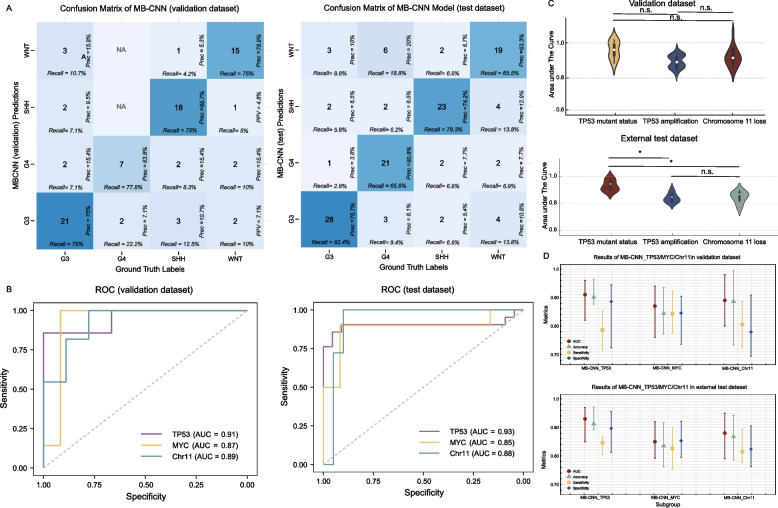
**A** Initial stage results of this study. The confusion matrix for the MB-CNN on validation and independent test datasets, used to assess the performance of a classification model. Each cell illustrates the relationship between actual and predicted categories, including number (the large number in each cell indicates how many times each category was predicted as another category) and precision (indicated by “P[rec] = X%” in each cell, it measures the proportion of correct predictions out of all predictions for that category). Recall (shown as “recall = X%” in each cell, it measures the proportion of actual instances of a category that were correctly predicted). **B** Second stage results of this study. The receiver operator characteristic (ROC) curve of classification effects of second stage (MB-CNN_TP53/MYC/Chr11) on the validation dataset and independent test dataset. **C** Violin plot of area under the curve (AUC) values for the MB-CNN_TP53/MYC/Chr11 to discriminate their corresponding gene signatures among different subgroups on validation and independent test dataset. **D** The dot plot of AUC, accuracy, sensitivity, and specificity of the second stage DL models (MB-CNN_TP53/MYC/Chr11) to discriminate their corresponding gene signatures among different subgroups on validation and independent test dataset

In the independent external test dataset, the model’s capability to distinguish between the MB molecular subgroups was further tested. Here, the model’s accuracy was in the range of 76.29% to 78.71%. Table 2 and Fig. 3A show the classification effect of different subgroups in the validation and external test datasets.

**Table 2:** Results of MB-CNN in validation and independent external test datasets and results of MB-CNN_TP53/MYC/Chr11 in different MB molecular subgroups

Subgroups	Accuracy [95% *CI*]	Recall [95% *CI*]	Precision [95% *CI*]	F1 score [95%*CI*]
**Validation dataset**				
WNT (*n* = 20)	74.68% [53.10%–88.88%]	75.00% [55.60%–93.30%]	78.90% [58.80%–95.01%]	76.90% [58.80%–90.09%]
SHH (*n* = 24)	75.00% [55.11%–88.25%]	74.69% [56.50%–92.67%]	85.70% [58.88%–95.00%]	80.00% [65.30%–91.29%]
Group 3 (*n* = 28)	76.29% [56.60%–87.31%]	76.48% [57.10%–90.00%]	75.00% [58.10%–90.50%]	75.00% [60.00%–86.20%]
Group 4 (*n* = 9)	77.84% [45.33%–93.70%]	77.81% [45.5%–100.00%]	73.85% [55.00%–84.67%]	63.60% [33.33%–83.33%]
**Independent external test dataset**				
WNT (*n* = 21)	76.29% [70.24%–92.34%]	75.86% [60.29%–91.44%]	78.75% [62.69%–84.81%]	72.13% [36.60%–72.84%]
SHH (*n* = 19)	78.71% [73.14%–94.28%]	82.76% [69.01%–96.51%]	72.73% [57.53%–87.92%]	77.42% [46.61%–81.53%]
Group 3 (*n* = 45)	77.90% [72.16%–93.64%]	76.47% [62.21%–90.73%]	78.79% [64.84%–92.74%]	77.61% [42.84%–75.86%]
Group 4 (*n* = 39)	77.10% [71.20%–92.99%]	65.63% [49.17%–82.08%]	80.77% [65.62%–95.92%]	72.41% [30.22%–64.82%]
	**AUC [95% CI]**	**Accuracy% [95% CI]**	**Sensitivity% [95% CI]**	**Specificity% [95% CI]**
**Validation dataset**				
SHH (TP53 mutant status) (*n* = 24)	0.911 [0.820–0.960]	90.14% [87.63%–96.42%]	78.62% [71.55%–85.44%]	88.57% [78.02%–94.43%]
G3 (MYC amplification) (*n* = 28)	0.870 [0.760–0.940]	84.43% [77.19%–93.53%]	84.32% [77.54%–92.42%]	84.54% [77.61%–90.43%]
G4 (chromosome 11 loss) (*n* = 9)	0.890 [0.800–0.980]	88.64% [73.42%–99.44%]	80.54% [72.55%–88.63%]	77.92% [69.41%–90.85%]
**Independent external test dataset**				
SHH (TP53 mutant status) (*n* = 19**)**	0.930 [0.850–0.970]	91.27% [89.34%–97.21%]	84.88% [80.23%–86.54%]	89.66% [81.42%–95.54%]
G3 (MYC amplification) (*n* = 45)	0.850 [0.740–0.920]	83.56% [76.23%–90.03%]	82.71% [75.44%–89.98%]	85.34% [79.52%–92.16%]
G4 (chromosome 11 loss) (*n* = 39)	0.880 [0.790–0.950]	86.79% [78.56%–94.22%]	81.47% [77.58%–89.36%]	82.38% [76.21%–90.55%]
*SHH* sonic hedgehog, *G3* Group 3, and *G4* Group 4 *AUC* area under the curve

### Results of the second stage: predicting prognostic-related genetic signatures in MB with MB-CNN_TP53/MYC/Chr11

In our analysis, we subdivided MB into distinct subgroups within the development dataset, each characterized by prognostic-related genetic signatures—TP53 gene mutation, MYC amplification, and chromosome 11 loss.

### Validation dataset analysis

Identified by TP53 gene mutation, this subgroup showed superior classification capabilities. The SHH subgroup exhibited an AUC of 0.91 [95% *CI*: 0.82–0.96] and a high accuracy of 90.14% [95% *CI*: 87.63%–96.42%].

Defined by MYC amplification, the G3 subgroup presented an AUC of 0.87 [95% *CI*: 0.76–0.94], with an accuracy of 84.43% [95% *CI*: 77.19%–93.53%].

Recognized by loss of chromosome 11, the G4 subgroup demonstrated an AUC of 0.89 [95% *CI*: 0.80–0.98] and accuracy of 88.64% [95% *CI*: 73.42%–99.44%].

### External test dataset analysis

Exhibiting remarkable classification performance, the SHH subgroup in the external test dataset, indicated by the TP53 mutation, showed an AUC of 0.93 [95% *CI*: 0.85–0.97] and an accuracy of 91.27% [95% *CI*: 89.34%–97.21%].

With MYC amplification, the G3 subgroup demonstrated an AUC of 0.85 [95% *CI*: 0.79–0.92] and an accuracy of 83.56% [95% *CI*: 76.23%–90.03%].

Characterized by chromosome 11 loss, the G4 subgroup exhibited an AUC of 0.88 [95% *CI*: 0.79–0.95] and an accuracy of 86.79% [95% *CI*: 78.56%–94.22%]. These results, highlighting the effectiveness of genetic signatures utilization for precise MB subgroup classification, are further detailed in Table 2 and Fig. 3B, showcasing the second stage model’s ability to discriminate among the subgroups based on their corresponding prognostic-related genetic signatures. Figure 3C and D shows the AUC values and accuracy, sensitivity, and specificity of MB-CNN_TP53/MYC/Chr11 in validation and independent external test datasets.

### Results of additional analysis: logistic regression and hybrid model

In our investigation, we developed a logistic regression model that integrated clinical parameters with features derived from MR radiographic assessment. Additionally, we synthesized a hybrid model by combining this logistic regression model with the outcomes from the MB-CNN. Both models underwent thorough evaluation on the independent external test dataset to ascertain their predictive efficacy.

**Fig. 4:**
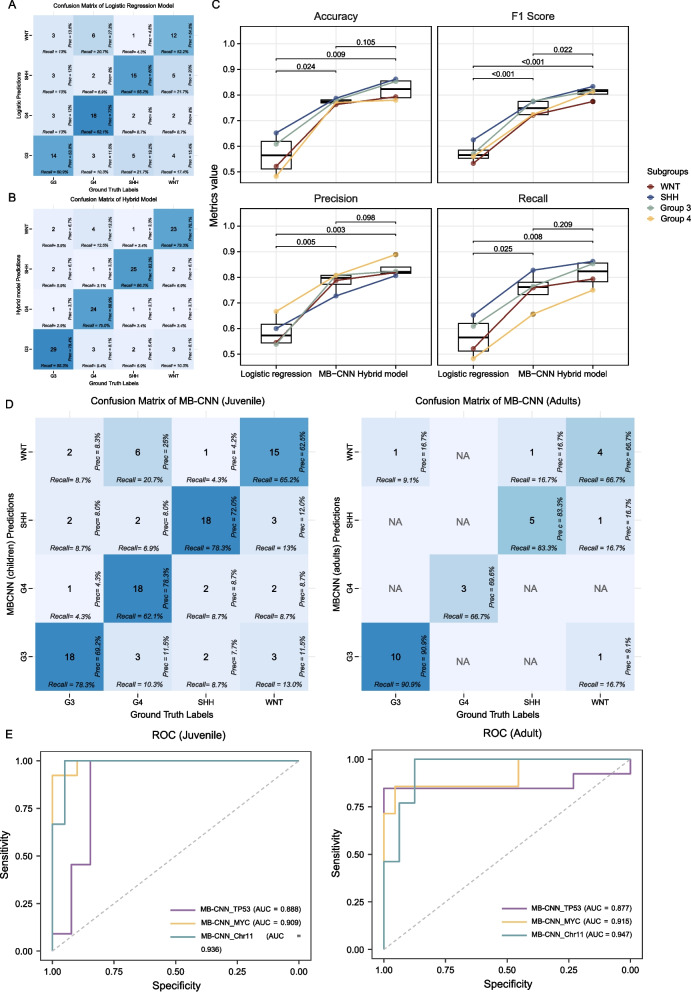
Additional analyses of this study. **A** The logistic regression model for confusion matrix results in the external test dataset. **B** The hybrid model for confusion matrix results in the external test dataset. **C** The performance metrics (accuracy, F1 score, precision, and recall) of the MB-CNN were systematically analyzed across various subgroup classifications of MB in the independent test dataset. **D** Additional analyses of this study. Confusion matrix results of the MB-CNN on the independent test dataset for the juvenile group (age ≤ 18 years) and adult group (age > 18 years). **E** The receiver operator characteristic (ROC) curve of classification effects of the second stage (MB-CNN_TP53/MYC/Chr11) on the independent test dataset for the juvenile group (age ≤ 18 years) and adult group (age > 18 years)

The logistic regression model, which utilized solely clinical and MR features, did not perform optimally. The model’s accuracy was in the range of 58.28% to 65.22%, with precision rates varying from 53.85% to 66.67% (Fig. 4A).

In contrast, the hybrid model displayed enhancements in performance metrics. Conversely, the hybrid model demonstrated improvements across various performance indicators. Its accuracy ranged between 78.00% and 86.21%, and precision varied from 75.86% to 88.89% (Fig. 4B). The comparative analysis revealed that the accuracy of the MB-CNN model demonstrated a mean enhancement of 21.04% relative to the baseline logistic regression. Further, the hybrid model exhibited an average accuracy improvement of 5.58% over the MB-CNN. These improvements highlight the efficacy of combining MB-CNN with clinical information and conventional MR features.

In a comparative analysis, the hybrid model was found to significantly outperform the logistic regression model (*P* = 0.009) and was competitively aligned with MB-CNN (*P* = 0.105), underscoring the advantageous potential of integrated approaches to enhance predictive precision within clinical settings. Table S2 details the classification effect of different models in the independent external test dataset. Figure 4C presents the growth rates of the different models.

### Subgroup analyses

We divided patients according to age into juvenile group (≤ 18 years) and adult group (> 18 years), and subgroup analysis was performed on data from the independent external test dataset. MB-CNN exhibited an accuracy of 68.97% to 85.67% and precision of 62.50% to 78.33% in the juvenile group. MB-CNN achieved an accuracy of 66.67% to 81.82% and precision of 69.57% to 78.33% in the adult group. Recall, F1 scores, and the increase rate of MB-CNN compared with logistic regression model and hybrid model compared with MB-CNN are presented in Table S3 and Fig. 4D. MB-CNN_TP53/MYC/Chr11 achieved AUC of 0.89 to 0.96, accuracy of 83.33% to 92.11% in juvenile group, and achieved AUC of 0.88 to 0.95, accuracy of 82.61% to 91.30% in adult group. The details of sensitivity and specificity are shown in Table S3 and Fig. 4E.

## Discussion

This study demonstrates the potential of deep learning models in classifying medulloblastoma subgroups and predicting prognostic genetic signatures, highlighting their utility in neuro-oncology diagnostics. Among the models tested, ResNet-50 outperformed others such as VGG, U-Net, and GoogLeNet for molecular subgroup classification, achieving a median accuracy of 77.50% (range 76.29–78.71%) and precision up to 80.77% on the external test dataset. The specialized MB-CNN_TP53/MYC/Chr11 models predicted high-risk genetic signatures with strong external validation performance, achieving AUC values of 0.93 for TP53 mutation in SHH, 0.85 for MYC amplification in Group 3, and 0.88 for chromosome 11 loss in Group 4. These metrics are detailed in Table 2. Compared to previous studies, such as Zhang et al. reporting ~ 70% accuracy for subgrouping and Chen et al. reporting ~ 85% without external validation, our model not only achieved comparable or higher classification performance but also extended predictions to clinically relevant genetic signatures. Furthermore, integrating DL outputs with clinical and MR data in a hybrid model improved diagnostic performance (accuracy: 82.20% vs. 59.14% for logistic regression alone, *P* = 0.009), illustrating the power of combining advanced machine learning with conventional data to refine medulloblastoma risk assessment and treatment planning.

Medulloblastoma, the most common pediatric malignant brain tumor, is divided into four molecular subgroups, each with distinct prognostic implications [12, 20, 21]. Advances in gene expression and DNA methylation profiling have expanded our understanding of MB pathogenesis, revealing molecular signatures that can inform therapy [4, 16, 17, 20]. However, identifying patients who may benefit from reduced intervention or those needing more intensive treatment remains a challenge [22]. Recognizing molecular subgroups is key to personalizing therapy, avoiding over- or under-treatment, and improving outcomes [23, 24]. While the WNT subgroup has a favorable prognosis, its molecular signatures do not significantly alter the clinical approach, underscoring the complexity of MB diagnostics [15, 25].

The integration of neuroimaging with machine learning holds significant promise for noninvasive molecular classification [23], enabling more precise therapeutic strategies. This synergy not only aids in preoperative identification of MB subgroups but also enhances risk monitoring [14, 15, 26]. Traditional diagnostic approaches, relying heavily on neuroradiologists’ experience, are limited by subjectivity and may misclassify subgroups [27]. Our study improves upon previous efforts by incorporating clinical, radiographic, and genetic data, significantly enhancing accuracy over prior models [28–30]. For example, while earlier work by Zhang et al. [15]and Chen et al. [20] showed promise, they lacked comprehensive data integration and had limited robustness [1, 3, 11, 31, 32]. By leveraging methylation and next-generation sequencing, our study introduces a more comprehensive and accurate approach to MB classification, offering a solid foundation for personalized treatment in clinical practice.

### Limitations

This study has several limitations. First, it was retrospective in nature and included patients from only two institutions, which may limit generalizability. Second, although we performed normalization to reduce inter-scanner variability, residual heterogeneity may remain due to differences in MRI protocols. Third, the sample size of adult patients, particularly within certain molecular subgroups such as WNT and Group 3, was small, which may limit the statistical power and generalizability of our findings in this cohort. Future studies with larger adult medulloblastoma datasets are warranted to further validate model performance. Finally, prospective validation and external testing in multicenter datasets are needed before clinical implementation.

## Conclusion

This study presents a deep learning framework utilizing MRI to predict four molecular subgroups and associated prognostic genetic signatures in medulloblastoma (MB). By integrating multi-semantic models and multidimensional data, we enhance the generalizability of AI in clinical contexts. While tissue specimens remain essential for diagnosis, this DL framework offers a cost-effective alternative or complement to traditional molecular risk assessments. Future work in imaging genomics and model deployment could expand personalized treatment strategies and inform clinical trial design.

## Supplementary Information

Supplementary Material 1: Supplementary Method 1. Dataset source, patient count, and allocation method. Supplementary Method 2. MRI parameters and contrast agent use. Supplementary Method 3. The details of the enhancement grading. Supplementary Method 4. Comprehensive molecular analysis of methylation patterns and genetic alterations in medulloblastoma. Supplementary Method 5. The comprehensive quality control protocol of utilization of MRI equipment from various manufacturers. Supplementary Method 6. Segmentation Model-Unet Network. Supplementary Method 7. Classification Model-ResNet 5. Supplementary Method 8. Additional details of statistical analysis. Supplementary Table 1. Comparison between different deep learning algorithms in the validation dataset. Supplementary Table 2. Results of logistic regression and hybrid model in the independent external test dataset. Supplementary Table 3. The performance of MB-CNN in juvenile group and adult group in subgroup analyses on independent test dataset. Supplementary Fig. 1. The proportion of manufactures in different datasets. Supplementary Fig. 2. The area under ROC curve, accuracy, sensitivity, and specificity of different algorithms.

## Data Availability

No datasets were generated or analysed during the current study.

## Abbreviations

DL: Deep learning
MB: Medulloblastoma
WNT: Wingless
SHH: Sonic hedgehog
G3: Group 3
G4: Group 4
AUC: Area under the receiver operating characteristic curve
Cis: Confidence intervals

## References

[1] Ramaswamy V, Remke M, Bouffet E, Bailey S, Clifford SC, Doz F, et al. Risk stratification of childhood medulloblastoma in the molecular era: the current consensus. Acta Neuropathol. 2016;131(6):821–31. 27040285 10.1007/s00401-016-1569-6 PMC4867119

[2] Louis DN, Perry A, Wesseling P, Brat DJ, Cree IA, Figarella-Branger D, et al. The 2021 WHO classification of tumors of the central nervous system: a summary. Neuro Oncol. 2021. 10.1093/neuonc/noab106. 34185076 10.1093/neuonc/noab106 PMC8328013

[3] Menyhart O, Gyorffy B. Molecular stratifications, biomarker candidates and new therapeutic options in current medulloblastoma treatment approaches. Cancer Metastasis Rev. 2020;39(1):211–33. 31970590 10.1007/s10555-020-09854-1 PMC7098941

[4] Gajjar A, Robinson GW, Smith KS, Lin T, Merchant TE, Chintagumpala M, et al. Outcomes by clinical and molecular features in children with medulloblastoma treated with risk-adapted therapy: results of an international phase III trial (SJMB03). J Clin Oncol. 2021;39(7):822–35. 33405951 10.1200/JCO.20.01372 PMC10166353

[5] Medulloblastoma. Nat Rev Dis Primers. 2019;5(1):12. 10.1038/s41572-019-0067-2 30765710

[6] Remke M, Ramaswamy V. WNT medulloblastoma limbo: how low can we go? Clin Cancer Res. 2022;28(19):4161–3. 35866882 10.1158/1078-0432.CCR-22-1780

[7] Chen L, Li Y, Song Z, Xue S, Liu F, Chang X, et al. O-glcnacylation promotes cerebellum development and medulloblastoma oncogenesis via SHH signaling. Proc Natl Acad Sci U S A. 2022;119(34): e2202821119. 35969743 10.1073/pnas.2202821119 PMC9407465

[8] Zhukova N, Ramaswamy V, Remke M, Pfaff E, Shih DJ, Martin DC, et al. Subgroup-specific prognostic implications of TP53 mutation in medulloblastoma. J Clin Oncol. 2013;31(23):2927–35. 23835706 10.1200/JCO.2012.48.5052 PMC4878050

[9] Shih DJ, Northcott PA, Remke M, Korshunov A, Ramaswamy V, Kool M, et al. Cytogenetic prognostication within medulloblastoma subgroups. J Clin Oncol. 2014;32(9):886–96. 24493713 10.1200/JCO.2013.50.9539 PMC3948094

[10] Fukuoka K, Kurihara J, Shofuda T, Kagawa N, Yamasaki K, Ando R, et al. Subtyping of group 3/4 medulloblastoma as a potential prognostic biomarker among patients treated with reduced dose of craniospinal irradiation: a Japanese pediatric molecular neuro-oncology group study. Acta Neuropathol Commun. 2023;11(1):153. 37749662 10.1186/s40478-023-01652-4 PMC10521425

[11] Ramaswamy V, Remke M, Adamski J, Bartels U, Tabori U, Wang X, et al. Medulloblastoma subgroup-specific outcomes in irradiated children: who are the true high-risk patients? Neuro Oncol. 2016;18(2):291–7. 25605817 10.1093/neuonc/nou357 PMC4724171

[12] Northcott PA, Buchhalter I, Morrissy AS, Hovestadt V, Weischenfeldt J, Ehrenberger T, et al. The whole-genome landscape of medulloblastoma subtypes. Nature. 2017;547(7663):311–7. 28726821 10.1038/nature22973 PMC5905700

[13] Sharma T, Schwalbe EC, Williamson D, Sill M, Hovestadt V, Mynarek M, et al. Second-generation molecular subgrouping of medulloblastoma: an international meta-analysis of group 3 and group 4 subtypes. Acta Neuropathol. 2019;138(2):309–26. 31076851 10.1007/s00401-019-02020-0 PMC6660496

[14] Chen X, Fan Z, Li KK, Wu G, Yang Z, Gao X, et al. Molecular subgrouping of medulloblastoma based on few-shot learning of multitasking using conventional MR images: a retrospective multicenter study. Neurooncol Adv. 2020;2(1):vdaa079. 10.1093/noajnl/vdaa079 PMC7393307 32760911

[15] Zhang M, Wong SW, Wright JN, Wagner MW, Toescu S, Han M, et al. MRI radiogenomics of pediatric medulloblastoma: a multicenter study. Radiology. 2022;304(2):406–16. 35438562 10.1148/radiol.212137 PMC9340239

[16] Leary SES, Packer RJ, Li Y, Billups CA, Smith KS, Jaju A, et al. Efficacy of carboplatin and isotretinoin in children with high-risk medulloblastoma: a randomized clinical trial from the Children’s Oncology Group. JAMA Oncol. 2021;7(9):1313–21. 34292305 10.1001/jamaoncol.2021.2224 PMC8299367

[17] Kumar R, Smith KS, Deng M, Terhune C, Robinson GW, Orr BA, et al. Clinical outcomes and patient-matched molecular composition of relapsed medulloblastoma. J Clin Oncol. 2021;39(7):807–21. 33502920 10.1200/JCO.20.01359 PMC8078396

[18] Savjani R. nnU-net: further automating biomedical image autosegmentation. Radiology: Imaging Cancer. 2021;3(1): e209039. 33778763 10.1148/rycan.2021209039 PMC7983725

[19] Konar D, Bhattacharyya S, Gandhi TK, Panigrahi BK, Jiang R. 3-D Quantum-inspired self-supervised tensor network for volumetric segmentation of medical images. IEEE Trans Neural Netw Learn Syst. 2024;35(8):10312-325. 10.1109/TNNLS.2023.3240238 37022399

[20] Schwalbe EC, Lindsey JC, Nakjang S, Crosier S, Smith AJ, Hicks D, et al. Novel molecular subgroups for clinical classification and outcome prediction in childhood medulloblastoma: a cohort study. Lancet Oncol. 2017;18(7):958–71. 28545823 10.1016/S1470-2045(17)30243-7 PMC5489698

[21] Coltin H, Sundaresan L, Smith KS, Skowron P, Massimi L, Eberhart CG, et al. Subgroup and subtype-specific outcomes in adult medulloblastoma. Acta Neuropathol. 2021;142(5):859–71. 34409497 10.1007/s00401-021-02358-4 PMC10723183

[22] Soomro TA, Zheng L, Afifi AJ, Ali A, Soomro S, Yin M, Gao J. Image segmentation for MR brain tumor detection using machine learning: a review. IEEE Rev Biomed Eng. 2023;16:70–90. 35737636 10.1109/RBME.2022.3185292

[23] Eran A, Ozturk A, Aygun N, Izbudak I. Medulloblastoma: atypical CT and MRI findings in children. Pediatr Radiol. 2010;40(7):1254–62. 20386894 10.1007/s00247-009-1429-9

[24] Dasgupta A, Gupta T, Maitre M, Kalra B, Chatterjee A, Krishnatry R, et al. Prognostic impact of semantic MRI features on survival outcomes in molecularly subtyped medulloblastoma. Strahlenther Onkol. 2022;198(3):291–303. 35059761 10.1007/s00066-021-01889-9

[25] Li J, Chen C, Fu R, Zhang Y, Fan Y, Xu J, Cen Y. Texture analysis of T1-weighted contrast-enhanced magnetic resonance imaging potentially predicts outcomes of patients with non-wingless-type/non-sonic hedgehog medulloblastoma. World Neurosurg. 2020;137:e27-33. 31589984 10.1016/j.wneu.2019.09.142

[26] Yan J, Liu L, Wang W, Zhao Y, Li KK, Li K, et al. Radiomic features from multi-parameter MRI combined with clinical parameters predict molecular subgroups in patients with medulloblastoma. Front Oncol. 2020;10: 558162. 33117690 10.3389/fonc.2020.558162 PMC7566191

[27] Alharbi M, Mobark N, Bashawri Y, Abu Safieh L, Alowayn A, Aljelaify R, et al. Methylation profiling of medulloblastoma in a clinical setting permits sub-classification and reveals new outcome predictions. Front Neurol. 2020;11:167. 32265819 10.3389/fneur.2020.00167 PMC7100767

[28] Magadza T, Viriri S. Deep learning for brain tumor segmentation: a survey of state-of-the-art. J Imaging. 2021. 10.3390/jimaging7020019. 34460618 10.3390/jimaging7020019 PMC8321266

[29] Richards BA, Lillicrap TP, Beaudoin P, Bengio Y, Bogacz R, Christensen A, et al. A deep learning framework for neuroscience. Nat Neurosci. 2019;22(11):1761–70. 31659335 10.1038/s41593-019-0520-2 PMC7115933

[30] Jayachandran Preetha C, Meredig H, Brugnara G, Mahmutoglu MA, Foltyn M, Isensee F, et al. Deep-learning-based synthesis of post-contrast T1-weighted MRI for tumour response assessment in neuro-oncology: a multicentre, retrospective cohort study. Lancet Digit Health. 2021;3(12):e784–94. 34688602 10.1016/S2589-7500(21)00205-3

[31] Waszak SM, Northcott PA, Buchhalter I, Robinson GW, Sutter C, Groebner S, et al. Spectrum and prevalence of genetic predisposition in medulloblastoma: a retrospective genetic study and prospective validation in a clinical trial cohort. Lancet Oncol. 2018;19(6):785–98. 29753700 10.1016/S1470-2045(18)30242-0 PMC5984248

[32] Luo Z, Xin D, Liao Y, Berry K, Ogurek S, Zhang F, et al. Loss of phosphatase CTDNEP1 potentiates aggressive medulloblastoma by triggering MYC amplification and genomic instability. Nat Commun. 2023;14(1):762. 36765089 10.1038/s41467-023-36400-8 PMC9918503

