# Supplementary material for: Exploring deep learning and hybrid approaches in molecular subgrouping and prognostic-related genetic signatures of medulloblastoma
Source: Chin Neurosurg J. 2025 Sep 15;11:19. doi: 10.1186/s41016-025-00405-7 (PMC12434915; doi:10.1186/s41016-025-00405-7)
Supplement: Supplementary file 1 — Supplementary Material 1: Supplementary Method 1. Dataset source, patient count, and allocation method. Supplementary Method 2. MRI parameters and contrast agent use. Supplementary Method 3. The details of the enhancement grading. Supplementary Method 4. Comprehensive molecular analysis of methylation patterns and genetic alterations in medulloblastoma. Supplementary Method 5. The comprehensive quality control protocol of utilization of MRI equipment from various manufacturers. Supplementary Method 6. Segmentation Model-Unet Network. Supplementary Method 7. Classification Model-ResNet 5. Supplementary Method 8. Additional details of statistical analysis. Supplementary Table 1. Comparison between different deep learning algorithms in the validation dataset. Supplementary Table 2. Results of logistic regression and hybrid model in the independent external test dataset. Supplementary Table 3. The performance of MB-CNN in juvenile group and adult group in subgroup analyses on independent test dataset. Supplementary Fig. 1. The proportion of manufactures in different datasets. Supplementary Fig. 2. The area under ROC curve, accuracy, sensitivity, and specificity of different algorithms. [file 41016_2025_405_MOESM1_ESM.docx]

**Supplementary Materials**

**Title: Exploring Deep Learning and Hybrid Approaches in Molecular Subgrouping and Prognostic Related Genetic Signatures of Medulloblastoma**

***Supplementary Method 1.*** Dataset source, patient count, and allocation method.

***Supplementary Method 2.*** MRI parameters and contrast agent use.

***Supplementary Method 3.*** The details of the enhancement grading.

***Supplementary Method 4.*** Comprehensive molecular analysis of methylation patterns and genetic alterations in medulloblastoma.

***Supplementary Method 5.*** The comprehensive quality control protocol of utilization of MRI equipment from various manufacturers.

***Supplementary Method 6.*** Segmentation Model-Unet Network.

***Supplementary Method 7.*** Classification Model-ResNet 50.

***Supplementary Method 8.*** Additional details of statistical analysis.

***Supplementary Table 1.*** Comparison between different deep learning algorithms in the validation dataset.

***Supplementary Table 2.*** Results of logistic regression and hybrid model in the independent external test dataset.

***Supplementary Table 3.*** The performance of MB-CNN in juvenile group and adult group in subgroup analyses on independent test dataset.

***Supplementary Figure 1.*** The proportion of manufactures in different datasets.

***Supplementary Figure 2.*** The area under ROC curve, accuracy, sensitivity and specificity of different algorithms.

***Supplementary Method 1.*** Dataset source, patient count, and allocation method.

The development dataset comprised 325 patients who were treated at Beijing Tiantan Hospital of the Capital Medical University from January 01, 2015, to June 30, 2023, and they were randomly divided into training and validation datasets according to the ratio of 3:1 (244:81). The independent external test dataset comprised 124 patients who were treated at Beijing Shijitan Hospital, Capital Medical University from January 2018, to June 30, 2023.

***Supplementary Method 2.*** MRI parameters and contrast agent use.

The T2W MR images parameters were as follows: repetition time / echo time = 3047-6711 / 84-119 ms; flip angle = 90°-160°; slice thickness = 3-6 mm; and matrix size = 256-328 × 512-512. T1W MR images comprised either three dimensional isovolumetric or two-dimensional spin-echo imaging, with repetition time / echo time = 17-30 / 4.3-4.6 ms; flip angle = 12°-15°; slice thickness = 0.8-5 mm; and matrix size = 240-256 × 384-512. For T1W imaging, contrast agents (gadolinium-based, CE-T1W), are used to increase the contrast between normal and pathological tissues by altering the relaxation times of tissues where they accumulate.

***Supplementary Method 3.*** The details of the enhancement grading.

Enhancements were classified as mild (slightly brighter than the surrounding tissue), moderate (showing a clearer contrast), or significant (where the lesion is distinctly pronounced). Additionally, they assessed for the presence of cerebellar origin, involvement of the brainstem, hemorrhage, cystic degeneration/necrosis, and signs of dissemination/metastasis.

***Supplementary Method 4.*** Comprehensive molecular analysis of methylation patterns and genetic alterations in medulloblastoma.

To ensure capturing the potential heterogeneity within a tumor and to provide more comprehensive information, we collect multiple samples from different parts of the tumor. By comparing tumor samples to normal cerebellar tissue, we first identified differentially methylated regions (DMRs) and selected genes at 75 percentiles in the absolute methylation difference for further analysis^1^. Using Principal Component Analysis (PCA), we explored the methylation patterns of these DMRs by generating a random β-value dataset (i.e., matching DMR β-values randomly with samples) and comparing the PCA results of this random data with the actual data to determine the number of principal components. To further reduce dimensionality and effectively visualize these complex methylation data, t-Distributed Stochastic Neighbor Embedding (t-SNE) technique was applied with specific parameter settings such as theta=0 to emphasize local data structures, pca=F to skip the initial PCA step, max_iter=2500 for the maximum number of iterations, and perplexity=10 to balance between the global and local data structures. Additionally, WGS was utilized to comprehensively screen for all potential mutation sites within the TP53 gene^2,3^, while CGH was employed to detect MYC gene amplification^4^ and loss of chromosome 11^5^.

In this study, we employed a comprehensive molecular analysis approach, specifically focusing on identifying differential methylation regions (DMRs) to accurately classify molecular subgroups of Medulloblastoma (MB). The criteria for selecting DMRs and determining the threshold for absolute methylation differences are crucial steps in this analysis, as they directly impact the accuracy and reliability of the molecular subgroup classification.

**Criteria for Selecting DMRs:**

Statistical Significance: Initially, we used statistical tests (such as the t-test) to identify methylation sites that showed significant differences between different MB molecular subgroups. Only sites with P-values less than 0.05 (after correction for multiple testing) were considered, ensuring that the identified DMRs were statistically significant.

Magnitude of Methylation Difference: Beyond statistical significance, we also set a minimum methylation difference threshold (for example, an absolute methylation difference ≥25%) to select biologically meaningful DMRs. This threshold was determined based on prior literature and preliminary experimental data, aiming to balance the identification of a sufficient number of DMRs while maintaining the specificity of the analysis.

Biological Function: Furthermore, we considered the biological function of the sites, prioritizing DMRs located in gene promoter regions or known to be associated with tumorigenesis and development. This helps ensure that the selected DMRs have potential biological relevance in the molecular mechanisms of MB.

**Determining the Threshold for Absolute Methylation Differences:**

The threshold for absolute methylation differences was set based on the extent of change in methylation status, aiming to distinguish clear differences between the molecular subgroups. In this study, an absolute methylation difference≥25% was used as the threshold for selecting DMRs. This threshold was determined based on several considerations:

Balance between Sensitivity and Specificity: The threshold needs to be high enough to ensure good discrimination between different subgroups by the identified DMRs, while also considering that too high a threshold might miss biologically important DMRs with smaller changes in methylation.

Data-Driven: By analyzing the methylation patterns in the training dataset, we evaluated the classification performance under different thresholds, ultimately choosing the threshold that maximized classification accuracy without significantly increasing the false-positive rate.

Literature and Previous Studies: Referencing thresholds commonly used in previous studies and literature in related fields, adjusted for our data characteristics and research objectives.

Through these criteria and threshold settings, we were able to effectively identify key DMRs related to the classification of MB molecular subgroups from complex methylation data, providing an accurate biological basis for subsequent deep learning model development and validation. This process ensures the scientific rigor and reliability of the method while enhancing the interpretability and applicability of the research findings.

***Supplementary Method 5.*** The comprehensive quality control protocol of utilization of MRI equipment from various manufacturers.

**Standardized Image Acquisition Protocol:**

Despite utilizing diverse equipment, we strived for uniformity in scanning parameters across all devices as much as possible, including echo time, repetition time, and flip angle. For parameters that could not be standardized, similar protocols were considered, or adjustments were made to minimize discrepancies.

**Image Preprocessing:**

Gaussian smoothing and background removal refined image quality for more accurate model learning. Data augmentation, such as rotation, flipping, and scaling, along with specific techniques like brain extraction and image registration, improved the model's generalization to unseen data. Cropping and resizing adjustments were made to meet the model's input requirements, optimizing computational efficiency and consistency. Feature extraction, utilizing both traditional and automated DL methods, was tailored to the analysis objectives, ensuring high-quality data input. The application of advanced data augmentation tools, such as those provided by Albumentations (*https://github.com/albumentations-team/albumentations*), further exemplifies the commitment to enhancing data quality and model efficacy.

Intensity Normalization: All MR images underwent intensity normalization to ensure a similar intensity distribution across the dataset^6^.

Spatial Standardization: Techniques such as resampling to a common space or applying standard brain atlases were used for spatial normalization.

Bias Field Correction: MR images were corrected for bias field or uneven brightness^7^.

**Data Augmentation:**

Data augmentation techniques, such as rotation, scaling, flipping, or adding noise, were applied to better training the model to handle images from different devices^8^.

**Cross-Device Validation:**

Cross-validation was performed using data from various MRI devices. For instance, data from one device was used for training and from another for testing.

Multicenter studies were conducted, including several centers using different MRI equipment^9^.

**Ensemble Learning Methods:**

Ensemble learning approaches were considered, combining predictions from multiple models corresponding to different devices to enhance overall performance and robustness.

***Supplementary Method 6.*** Segmentation Model-Unet Network.

**Model Architecture**

The Unet model, a prominent architecture in medical image analysis, employs an Encoder-Decoder framework^10,11^. This structure is critical for capturing intricate details in medical images, particularly in the context of brain tumor morphology. The Encoder stage involves consecutive convolutional and max pooling layers, enabling the extraction of high-dimensional features. The Decoder stage then reconstructs the segmentation map through up-convolutional layers, merging them with the corresponding encoder features.

In this application, Unet segments the original MRI to delineate the precise shape and spatial location of brain tumors. This segmentation is crucial for accurate subsequent genetic subtyping.

**Segmentation Dataset**

Comprised of MRI scans from 325 patients, covering three specific sequences (T1W, contrast enhanced T1W, T2W) to optimize tumor feature extraction. The dataset split is 80% for training, 10% for validation, and 10% for testing.

**Segmentation Training**

The Unet model is trained on the PyTorch platform for 100 epochs. The Adam optimizer is employed, along with a cosine decay schedule for the learning rate. The Dice Loss function is used as the loss metric, and the model's performance is periodically evaluated using the Dice Score on the validation set.

***Supplementary Method 7.*** Classification Model-ResNet 50.

**Model Architecture**

The ResNet 50 model^12-14^, known for its deep residual learning framework, consists of 50 layers. Its architecture is designed to combat the vanishing gradient problem in deep networks. The residual blocks facilitate the flow of gradients, enabling efficient training of deeper networks.

Application: In the classification stage, ResNet 50 is utilized to categorize the tumor into one of four genetic subtypes. The model processes the combined input of original MRI images and Unet-derived tumor segmentation maps. The images are resized to 256x256x3 and undergo convolutional transformations within ResNet 50, followed by a final classification via a fully connected layer.

**Classification Dataset**

Utilizes the same 325 patient dataset. Each entry includes the original MRI scan and its corresponding tumor segmentation map from the Unet model. The dataset split ensures balanced representation across the four molecular subtypes.

**Classification Training**

ResNet 50 undergoes training for 10 epochs on PyTorch. The FocalLoss function is utilized for addressing class imbalance. Model performance is assessed based on accuracy metrics on the validation and external test dataset.

***Supplementary Method 8.*** Additional details of statistical analysis.

The Dice score, defined as the spatial overlap between different raters or the ground truth and DL segmentation, was used for the assessment of tumor segmentation performance. The performances of DL model and neuroradiologists were assessed using accuracy, specificity, sensitivity, and AUC. Confidence intervals (CIs) for these parameters were calculated using bootstrapping with 1000 iterations to obtain 95% CIs.

***Supplementary Table 1.*** Comparison between different deep learning algorithms in the validation dataset.

| **Algorithms** | **Accuracy [95%CI]** | **Recall [95%CI]** | **Precision [95%CI]** | **F1 Score [95%CI]** |
| --- | --- | --- | --- | --- |
| VGG | 65.43% [47.77%-71.00%] | 68.40% [62.40%-77.10%] | 65.03% [47.28%-71.66%] | 72.55% [62.42%-77.35%] |
| Unet | 72.11% [62.01%-82.44%] | 74.11% [65.40%-84.30%] | 72.00% [62.78%-82.33%] | 75.43% [65.33%-84.32%] |
| GoogleNet | 69.56% [55.22%-79.66%] | 77.42% [67.42%-83.24%] | 69.06% [55.43%-79.87%] | 71.67% [66.98%-80.41%] |
| ResNet 50 | 79.41% [69.43%-86.54%] | 73.65% [55.64%-82.32%] | 78.05% [69.00%-86.33%] | 70.63% [64.32%-81.64%] |

***Supplementary Table 2.*** Results of logistic regression and hybrid model in the independent external test dataset.

| **Subgroups** | **Metrics** | **Logistic regression model** | **MB-CNN** | **Increase rate  (Logistic regression vs. MB-CNN)** | **Hybrid model** | **Increase rate  (MB-CNN vs. Hybrid model)** |
| --- | --- | --- | --- | --- | --- | --- |
| **WNT** | Accuracy | 52.17% [31.76% - 72.59%] | 76.29% [70.24% - 92.34%] | 31.61% | 79.31% [64.57% - 94.05%] | 3.81% |
| (n=21) | Precision | 54.55% [33.74% - 75.35%] | 78.75% [62.69% - 84.81%] | 30.73% | 81.86% [71.54% - 91.30%] | 3.79% |
|  | Recall | 52.17% [31.76% - 72.59%] | 75.86% [60.29% - 91.44%] | 31.23% | 79.33% [60.00% - 92.59%] | 4.37% |
|  | F1 Score | 53.33% [33.31% - 69.23%] | 72.13% [36.60% - 72.84%] | 26.06% | 77.46% [63.16% - 86.96%] | 6.88% |
| **SHH** | Accuracy | 65.22% [45.75% - 84.68%] | 78.71% [73.14% - 94.28%] | 17.14% | 86.21% [73.66% - 98.76%] | 8.70% |
| (n=19) | Precision | 60.00% [40.80% - 79.20%] | 72.73% [57.53% - 87.92%] | 17.50% | 80.65% [66.67% - 95.65%] | 9.82% |
|  | Recall | 65.22% [45.75% - 84.68%] | 82.76% [69.01% - 96.51%] | 21.19% | 86.21% [72.00% - 96.88%] | 4.00% |
|  | F1 Score | 62.50% [43.75% - 76.19%] | 77.42% [46.61% - 81.53%] | 19.27% | 83.33% [71.99% - 93.03%] | 7.09% |
| **Group 3** | Accuracy | 60.87% [40.92% - 80.81%] | 77.90% [72.16% - 93.64%] | 21.86% | 85.29% [73.39% - 97.20%] | 8.66% |
| (n=45) | Precision | 53.85% [34.68% - 73.01%] | 81.74% [67.42% - 93.11%] | 33.33% | 82.38% [65.00% - 91.49%] | 1.95% |
|  | Recall | 60.87% [40.92% - 80.81%] | 76.47% [62.21% - 90.73%] | 20.40% | 85.29% [72.50% - 96.97%] | 10.34% |
|  | F1 Score | 57.14% [38.89% - 72.73%] | 77.61% [42.84% - 75.86%] | 26.38% | 81.69% [71.18% - 90.91%] | 4.99% |
| **Group 4** | Accuracy | 58.28% [40.09% - 66.46%] | 77.10% [71.20% - 92.99%] | 24.41% | 78.00% [60.00% - 90.00%] | 1.15% |
| (n=39) | Precision | 66.67% [46.50% - 86.83%] | 80.77% [65.62% - 95.92%] | 17.46% | 88.89% [76.00% -100.00%] | 9.13% |
|  | Recall | 48.28% [30.09% - 66.46%] | 65.63% [49.17% - 82.08%] | 26.44% | 75.00% [59.24% - 89.66%] | 12.49% |
|  | F1 Score | 56.00% [36.36% - 71.11%] | 72.41% [30.22% - 64.82%] | 22.66% | 81.36% [69.39% - 91.23%] | 11.00% |

***Supplementary Table 3.*** The performance of MB-CNN in juvenile group and adult group in subgroup analyses on independent test dataset.

|  | **Accuracy [95%CI]** | **Recall [95%CI]** | **Precision [95%CI]** | **F1 Score [95%CI]** |
| --- | --- | --- | --- | --- |
| **Juvenile group (**≤ **18 years)** | | | | |
| WNT (n=18) | 78.91% [55.97% - 91.86%] | 69.88% [56.52% - 91.30%] | 62.50% [56.52% - 91.30%] | 76.83% [57.14% - 85.71%] |
| SHH (n=11) | 85.67% [67.12% - 98.10%] | 79.81% [65.22% - 95.65%] | 72.01% [60.00% - 92.00%] | 78.92% [64.86% - 90.57%] |
| Group 3 (n=40) | 82.61% [55.43% - 88.92%] | 83.66% [67.11% - 98.41%] | 78.33% [60.85% - 82.43%] | 79.17% [63.82% - 89.80%] |
| Group 4 (n=29) | 68.97% [52.13% - 85.80%] | 77.63% [51.72% - 86.21%] | 69.20% [64.00% - 86.00%] | 74.07% [59.26% - 85.19%] |
| **Adult group (> 18 years)** | | | | |
| WNT (n=3) | 74.67% [58.95% - 84.39%] | 82.11% [28.95% - 100.00%] | 66.77% [58.33% - 96.00%] | 71.21% [55.43% - 83.42%] |
| SHH (n=8) | 79.33% [61.42% - 84.66%] | 80.01% [53.51% - 100.00%] | 83.31% [62.86% - 100.00%] | 76.92% [66.44% - 82.11%] |
| Group 3 (n=5) | 81.82% [69.03% - 97.61%] | 75.63% [59.03% - 100.00%] | 90.90% [79.00% - 100.00%] | 85.71% [76.44% - 93.63%] |
| Group 4 (n=10) | 66.67% [53.32% - 75.65%] | 66.67% [43.32% - 81.64%] | 69.57% [57.88% - 85.42%] | 69.77% [61,65% - 83.67%] |
|  | **AUC [95%CI]** | **Accuracy [95%CI]** | **Sensitivity [95%CI]** | **Specificity [95%CI]** |
| **Juvenile group (**≤ **18 years)** | | | | |
| MB-CNN_TP53 (n=11) | 0.888 [0.745 - 1.000] | 83.33% [66.67% - 95.83%] | 81.82% [54.55% - 100.00%] | 84.62% [61.54% - 100.00%] |
| MB-CNN_MYC (n=40) | 0.909 [0.796 - 0.950] | 86.96% [73.91% - 100.00%] | 84.62% [61.54% - 100.00%] | 90.00% [70.00% - 100.00%] |
| MB-CNN_Chr11 (n=29) | 0.936 [0.856 - 1.000] | 92.11% [81.58% - 100.00%] | 88.89% [72.22% - 100.00%] | 95.00% [85.00% - 100.00%] |
| **Adult group (>18 years)** | | | | |
| MB-CNN_TP53 (n=8) | 0.877 [0.689 - 1.000] | 82.61% [65.22% - 95.65%] | 80.00% [53.85% - 100.00%] | 84.62% [62.50% - 100.00%] |
| MB-CNN_MYC (n=5) | 0.915 [0.770 - 1.000] | 86.96% [69.57% - 100.00%] | 84.62% [61.54% - 100.00%] | 90.00% [66.67% - 100.00%] |
| MB-CNN_Chr11 (n=10) | 0.947 [0.810 - 1.000] | 91.30% [78.26% - 100.00%] | 91.67% [73.33% - 100.00%] | 90.91% [69.23% - 100.00%] |

***Supplementary Figure 1.*** The proportion of manufactures in different datasets.


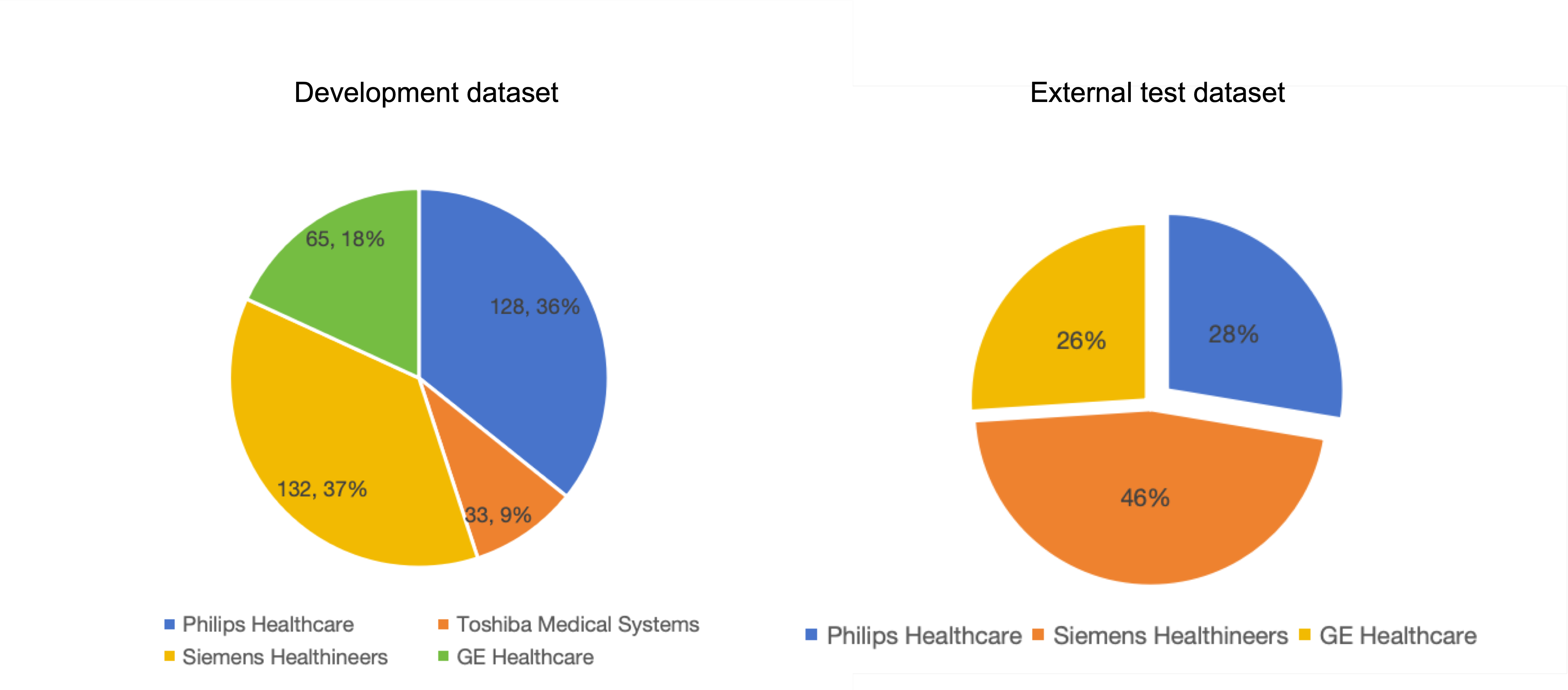


***Supplementary Figure 2.*** The area under ROC curve, accuracy, sensitivity, and specificity of different algorithms.


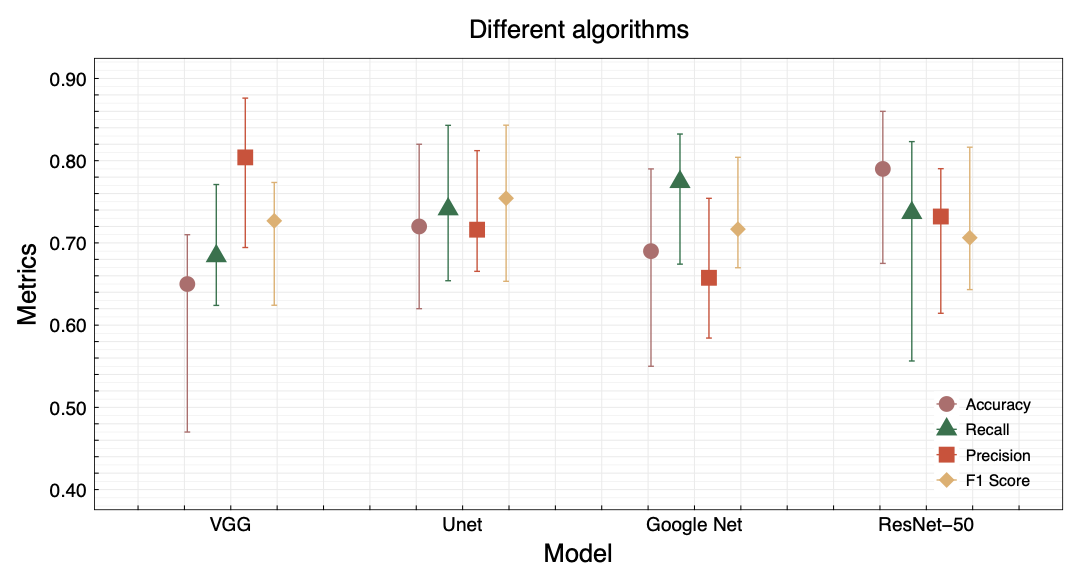


**References**

**1.** Davies MN, Volta M, Pidsley R, et al. Functional annotation of the human brain methylome identifies tissue-specific epigenetic variation across brain and blood. *Genome Biol.* 2012; 13(6):R43.

**2.** Zhukova N, Ramaswamy V, Remke M, et al. Subgroup-specific prognostic implications of TP53 mutation in medulloblastoma. *J Clin Oncol.* 2013; 31(23):2927-2935.

**3.** Eibl RH, Schneemann M. Medulloblastoma: From TP53 Mutations to Molecular Classification and Liquid Biopsy. *Biology (Basel).* 2023; 12(2).

**4.** Kumar D, Jain S, Coulter DW, Joshi SS, Chaturvedi NK. PRMT5 as a Potential Therapeutic Target in MYC-Amplified Medulloblastoma. *Cancers (Basel).* 2023; 15(24).

**5.** Ramaswamy V, Remke M, Bouffet E, et al. Risk stratification of childhood medulloblastoma in the molecular era: the current consensus. *Acta Neuropathol.* 2016; 131(6):821-831.

**6.** Lundervold AS, Lundervold A. An overview of deep learning in medical imaging focusing on MRI. *Z Med Phys.* 2019; 29(2):102-127.

**7.** Thamm M, Jeffery JJ, Zhang Y, et al. Multimodal Cross-Device and Marker-Free Co-Registration of Preclinical Imaging Modalities. *J Vis Exp.* 2023(200).

**8.** Hao R, Namdar K, Liu L, Haider MA, Khalvati F. A Comprehensive Study of Data Augmentation Strategies for Prostate Cancer Detection in Diffusion-Weighted MRI Using Convolutional Neural Networks. *J Digit Imaging.* 2021; 34(4):862-876.

**9.** Artan Y, Oto A, Yetik IS. Cross-device automated prostate cancer localization with multiparametric MRI. *Annu Int Conf IEEE Eng Med Biol Soc.* 2012; 2012:6247-6250.

**10.** Falk T, Mai D, Bensch R, et al. U-Net: deep learning for cell counting, detection, and morphometry. *Nat Methods.* 2019; 16(1):67-70.

**11.** Savjani R. nnU-Net: Further Automating Biomedical Image Autosegmentation. *Radiol Imaging Cancer.* 2021; 3(1):e209039.

**12.** Emam MM, Samee NA, Jamjoom MM, Houssein EH. Optimized deep learning architecture for brain tumor classification using improved Hunger Games Search Algorithm. *Comput Biol Med.* 2023; 160:106966.

**13.** Asad R, Rehman SU, Imran A, Li J, Almuhaimeed A, Alzahrani A. Computer-Aided Early Melanoma Brain-Tumor Detection Using Deep-Learning Approach. *Biomedicines.* 2023; 11(1).

**14.** Ahmad B, Sun J, You Q, Palade V, Mao Z. Brain Tumor Classification Using a Combination of Variational Autoencoders and Generative Adversarial Networks. *Biomedicines.* 2022; 10(2).
